# Lumican Inhibits SNAIL-Induced Melanoma Cell Migration Specifically by Blocking MMP-14 Activity

**DOI:** 10.1371/journal.pone.0150226

**Published:** 2016-03-01

**Authors:** Marta Stasiak, Joanna Boncela, Corinne Perreau, Konstantina Karamanou, Aurore Chatron-Colliet, Isabelle Proult, Patrycja Przygodzka, Shukti Chakravarti, François-Xavier Maquart, M. Anna Kowalska, Yanusz Wegrowski, Stéphane Brézillon

**Affiliations:** 1 CNRS UMR 7369, Matrice Extracellulaire et Dynamique Cellulaire (MEDyC), Université de Reims Champagne Ardenne, Laboratoire de Biochimie Médicale et de Biologie Moléculaire, Reims, France; 2 Department of Cytobiology and Proteomics, Medical University of Lodz, Lodz, Poland; 3 Institute of Medical Biology, Polish Academy of Sciences, Lodz, Poland; 4 Laboratory of Biochemistry, Department of Chemistry, University of Patras, Patras, Greece; 5 Department of Medicine, Johns Hopkins University School of Medicine, Baltimore, MD, United States of America; 6 CHU de Reims, Laboratoire Central de Biochimie, Reims, France; 7 Division of Hematology, The Children’s Hospital of Philadelphia, Philadelphia, PA, United States of America; University of Crete, GREECE

## Abstract

Lumican, a small leucine rich proteoglycan, inhibits MMP-14 activity and melanoma cell migration *in vitro* and *in vivo*. Snail triggers epithelial-mesenchymal transitions endowing epithelial cells with migratory and invasive properties during tumor progression. The aim of this work was to investigate lumican effects on MMP-14 activity and migration of Snail overexpressing B16F1 (Snail-B16F1) melanoma cells and HT-29 colon adenocarcinoma cells. Lumican inhibits the Snail induced MMP-14 activity in B16F1 but not in HT-29 cells. In Snail-B16F1 cells, lumican inhibits migration, growth, and melanoma primary tumor development. A lumican-based strategy targeting Snail-induced MMP-14 activity might be useful for melanoma treatment.

**Highlights**

Snail stimulates MMP-14 activity in Snail overexpressing B16F1 melanoma cells but not in HT29 cells; Lumican inhibits the Snail-induced MMP-14 activity in Snail-B16F1 cells; Lumican inhibits the migration and growth of Snail-B16F1 cells *in vitro*; Lumican inhibits melanoma primary tumor growth of Snail-B16F1 cells *in vivo*.

## Introduction

Lumican belongs to the family of the small leucine–rich proteoglycans (SLRP) that contribute to extracellular matrix (ECM) assembly and organization through protein:protein and/or protein:carbohydrate interactions [1–3]. It is expressed widely in mesenchymal connective tissues [4, 5] and present as a proteoglycan in some tissues like the cornea due to post-translational addition of keratan sulfate glycosaminoglycan side chains, or simply as a glycoprotein in some tissues [6, 7]. Lumican’s leucine rich repeat (LRR) motifs [7–9] participate in the process of collagen assembly [10], control cell migration [11] and also regulate tumor cell progression [12, 13].

Data from our laboratory demonstrated anti-tumorigenic properties of lumican in melanoma both *in vitro* and *in vivo* [14–18]. Lumican core protein (37 kDa) was reported to increase melanoma cell adhesion [16] and the glycosylated protein (57 kDa) inhibited melanoma cell proliferation, migration and invasion [14, 17, 18]. The expression of lumican is preferentially located at the periphery of the melanoma tumor in stromal dermal fibroblasts [15]. Its expression in melanoma cells was dependent on the cell line used [15, 19]. In a mouse melanoma model of B16F1 injection, tumor formation was significantly inhibited when human lumican overexpressing cells were used. Lumican inhibits melanoma cell migration by alteration of the actin network and focal adhesion complexes [17, 20, 21] and this process is mediated by α2β1 integrin that binds lumican directly [18]. In addition, it was shown that lumican had angiostatic properties and inhibited lung metastatic nodules in mice [11, 22–24]. The lumican inhibitory effect on the migration of endothelial cells is associated with regulation of the expression and activity of MMP-9 and MMP-14 *via* integrins [24]. MMP-14 plays an important role in cell migration not only by regulating the activity or expression of downstream MMPs, but also by processing and activating migration-associated molecules such as integrins and a variety of intracellular signaling pathways [25]. In approximately 63% of colorectal cancer patients, lumican is up regulated [26]. Lumican was also localized in epithelial cells with mild reactive dysplasia and fibroblasts adjacent to colon cancer cells. These findings indicate that the lumican synthesized by cancer cells, fibroblasts and epithelial cells may affect the growth of human colorectal cancer [27]. Overexpression of lumican has also been shown to affect the migration of human colon cancer cells through up regulation of gelsolin and filamentous actin reorganization [20, 21].

MMPs are overexpressed in various human malignancies and have been thought to contribute to tumor invasion and metastasis by degrading ECM components [28, 29]. Considering the important impact of MMP-14 in tumor cell migration and malignant progression and the anti-migratory and anti-tumorigenic role of lumican (for review see [12]), we focused on the direct interaction between these two macromolecules. We recently showed that the glycosylated form of lumican was able to significantly decrease MMP-14 activity in B16F1 melanoma cells [30]. While MMP-14 plays a critical role in melanoma progression, its overexpression in colon adenocarcinoma cells was reported to be insufficient to increase experimental liver metastasis of human colon cancer cells [31].

Snail is one of the major transcription factors governing epithelial-mesenchymal transition (EMT) of various cancer cells, and its increase in tumor tissues of patients is correlated with tumor progression (metastasis and recurrence) in various cancers including melanoma [32–34], hepatocellular carcinoma [35], head and neck squamous cell carcinoma [36], and endometrial cancers [37]. In EMT and melanoma progression, the underlying mechanism is a disruption in growth control of keratinocytes due to Snail-mediated downregulation of E-cadherin [38]. Thus, the loss of this epithelial marker, a hallmark of EMT in carcinoma, was observed in late-stage melanoma that invariably metastasized [39–41]. Kudo-Saito and collaborators demonstrated that Snail-induced EMT accelerated melanoma metastasis through not only enhanced invasion but also induction of immunosuppression [42]. Their results suggest that inhibition of Snail-induced EMT could simultaneously suppress tumor metastasis and lift immunosuppression in cancer patients.

While aberrant reactivation of EMT in epithelial cells was described to be oncogenic, the functions of EMT-inducing transcription factors, like Snail, in non-epithelial cells remain poorly understood [41]. Since malignant melanoma represents one of the deadliest cancer types at the metastatic stage, the aim of the study was to investigate the effect of lumican on MMP-14 activity and migration capacities of Snail overexpressing melanoma cells.

## Materials and Methods

### Materials

Recombinant human pro-MMP-14 (catalytic domain, amino acids 89–265) was obtained from Merck Millipore (Nottingham, UK). Prior to the enzymatic activity assays, pro-MMP-14 was incubated with APMA (AnaSpec, San Jose, USA) to convert the enzyme in the active form. Recombinant human lumican (57 kDa) and its core protein (37 kDa) were produced as previously described [14, 18] or purchased from R&D Systems (#2846-LU-050, R&D Systems, MN, USA). Rabbit polyclonal anti-lumican antibody was produced as previously described [14]. Secondary antibodies conjugated to horseradish peroxidase (HRP) were purchased from GE Healthcare (Orsay, France) or from Santa Cruz Biotechnology (Dallas, TX, USA).

### Cell culture

Murine B16F1 melanoma cells from ATCC (CRL-6323^™^) were cultured in DMEM in standard conditions [14]. HT29 colorectal adenocarcinoma cells (HTB-38^™^, ATCC) were cultured in McCoy’s 5A (Gibco^™^, Invitrogen) supplemented with 10% FBS and 1% penicillin/streptomycin. In all experiments, cell viability was greater than 95% as assessed by trypan blue exclusion test.

### Vector construction and transfection of human *SNAIL* cDNA

The cell expression construct (pcDNA3.1- human *SNAIL*) was obtained from Prof. Muh-Hwa Yang (Taipei Veterans General Hospital, Taiwan). Cells were grown to 85% confluence and transfected with 5 μg DNA/10^6^ cells using the Amaxa^®^ 4D nucleofector ^®^ X Unit (Lonza, Basel, Switzerland) according to the manufacturer’s instructions. Subsequently, the cells were cultured in medium supplemented with 200 μg/mL G418/Geneticin (Gibco/LifeTechnologies, Waltham, MA, USA). The selection medium was refreshed every 48 h. After 2 weeks in culture, well-separated colonies were isolated. The culture of clones was scaled-up and *SNAIL*expression was verified through real-time PCR and Western blot analysis. Mock (transfected with the empty plasmid) -B16F1 and -HT29 stable clones were also isolated by critical dilution in presence of 200 μg/ml of G418, screened and served as controls.

### RNA isolation and RT-PCR analysis

#### B16F1 cells

Total RNA of wild-type, Mock- and Snail overexpressing B16F1 cells (Snail-B16F1) was isolated using RNeasy^®^ Plus Mini Kit (Qiagen, Courtaboeuf, France) according to manufacturer's instructions. Determination of RNA quality was performed on an Agilent 2100 Bioanalyzer (Agilent Technologies, Massy, France). Reverse transcription was performed at 50°C for 30 min with 1 μg of total RNA and the Maxima First Strand cDNA synthesis kit with double strand DNAse (Thermo Scientific, Villebon sur Yvette, France). Real-time PCR experiments were performed on an Mx3005P thermocycler (Agilent Technologies, Massy, France) with Maxima SYBR Green/ROX qPCR Master Mix (Thermo Scientific) and specific primers for human *SNAIL* and *MMP-14*. Primer sequences and sizes of the PCR product for each targeted gene are described in supplemental material (S1 Table). Each sample was normalized simultaneously to *EF1a* and *GAPDH* housekeeping gene transcript content. The ΔΔCt method was used for the relative quantification. PCR assays were conducted in triplicate for each sample.

#### HT29 cells

Total RNA of wild-type, Mock- and Snail overexpressing HT29 cells (Snail-HT29) was isolated from cells using miRCURY^™^ RNA Isolation Kit (Exiqon, Denmark) according to manufacturer’s instructions. TaqMan Gene Expression Assay for *SNAIL*(HS00195591_m1) was used with the TaqMan Universal PCR master mix and the ABI Prism7900-HT detection system (Applied Biosystems, Foster City, CA, USA). *GADPH* and *β-actin* mRNA transcripts were used as internal control genes. The amount of target mRNA in the various samples was estimated using the 2^-ΔΔCT^ or the 2^-ΔCT^ relative quantification method with DataAssist v.3.01 software.

### Immunoblotting

Cell monolayers were washed, scrapped and lysed in buffer [50 mM Tris-HCl (pH 7.6), 0.5 M NaCl, 0.02% NaN_3_, 0.6% NP40, 5 mM EDTA, 1 mM iodoacetamide, 1 mM PMSF]. The protein concentration was determined by Bradford method [43]. When needed, the protein extracts were incubated for 24 h at 37°C with 100 nM lumican. Total cell proteins (10 μg) were subjected to electrophoresis in a 0.1% SDS, polyacrylamide gel and proteins were transferred onto PVDF membranes (Millipore). The membranes were blocked in 5% non fat milk (BioRad) for 2h at RT, washed with TBS/0.1% Tween 20 and the membranes were incubated overnight at 4°C with the following primary antibodies: anti-human Snail (#3879, Cell Signaling Technology, 1:500), anti-actin (#sc-1616, Abcam, 1:500) and anti-MMP-14 (#ab38971, Abcam, 1:5000). After washing and incubation with corresponding HRP-secondary antibody, bands were visualized using the ECL Plus Chemoluminescence Detection kit (GE Healthcare, Orsay, France or Thermo Scientific, Waltham, MA, USA). Membranes were either developed with Kodak BioMax Light Film (Eastman Kodak, Rochester, NY, USA) or scanned on a Chemidoc (Biorad, Marne la coquette, France) imaging and gel documentation system. The protein extracts and the control lysate from human *SNAIL* transfected 293T cells (Santa Cruz Biotechnology) were used as a control.

### MMP-14 activity assay

The MMP-14 activity was measured in 96-well plates using 1 μM of the fluorogenic substrate: 5-FAM/QXL^™^520 FRET peptide in the reaction buffer supplied in SensoLyte^®^ 520 MMP-14 Assay Kit (AnaSpec, San Jose, USA) at excitation and emission wavelengths of 490 and 520 nm, respectively. The assays were carried out in triplicate at 37°C. Fluorescence was measured with a spectrofluorometer (Mithras LB940, Berthold Technologies, Thoiry, France). The MMP-14 activity of HT-29 and B16F1 cell lysates was measured *in vitro* after 24h of incubation of the cells without or with 100 nM lumican. Results are the average of two and three independent experiments for Snail8-HT29 and for Snail19-B16F1, respectively.

### Gelatin zymography

To determine MMP-2 and MMP-9 activities, mock or Snail overexpressing HT-29 and B16F1 cell-conditioned concentrated media were analyzed on SDS-polyacrylamide gels containing 1 mg/ml gelatin. When needed, cells were incubated 24h at 37°C with 100 nM lumican. Recombinant MMP-2 and MMP-9 (Millipore) were used as markers. The gels were stained with Coomassie Brillant Blue G-250 (Sigma) and MMP activities were detected as transparent bands on the blue background.

### *In vitro* wound healing-like migration assays

The migration assay was performed using culture-inserts (Biovalley, Marne-la-Vallée, France) composed of 2 chambers separated by a “wall”. After withdrawing of the insert, the empty space left by the “wall” simulates a wound and enables the cells to migrate. Cells were seeded on 24-well plates in culture-inserts at a concentration of 5×10^4^ cells/ml (70 μl of cell suspension per chamber). Twenty four hours after incubation at 37°C, the culture inserts were removed, cells were rinsed twice with PBS and the wells were filled with 2 ml of serum-free cell culture medium. When needed, lumican (57 kDa) was added at a final concentration of 100 nM. The wound healing was followed for 48h with photos taken every 24h using an inverted microscope (Axiovert 200M; Zeiss, Oberkoken, Germany) equipped with a digital camera. Wound closure was estimated from 3 independent experiments by taking pictures of 3 microscopic fields per insert, 3 replicate inserts for each condition. The average wound area was measured by Image J software.

### Anchorage-independent growth

Anchorage-independent soft agar growth assays were carried out in 24-well plates. Each well contained the following layers: a bottom layer of 0.9% agar (400 μl), a middle layer of 0.3% agar (1 ml) containing the cell suspension (2.5 x 10^3^ cells/well) and a top layer of 0.9% agar (400 μl). Agar was previously mixed 1:1 with 2 x growth medium (DMEM medium supplemented with 5% FBS). After 14 days of culture, the number of the colonies was counted and the size of colonies was measured in triplicate [14, 44].

### Generation and maintenance of lumican deficient mice

The Lumican deleted (*Lum*^-/-^) mouse line was generated by targeted gene disruption as previously described [4]. All animals used in this study were of a C57BL/6 genotype background and were genotyped by PCR. All mice procedures conformed to the ethical rules of The University of Reims Champagne Ardenne ethical committee and were approved by URCAnim REIMS EU0362, and the CNRS (Protocol number: 56-2012-14).

### *In vivo* tumor growth studies

Wild-type C57BL/6 mice and *Lum*^-/-^ C57BL/6 mice [4] were subcutaneously injected in the right flank with 2.5 x 10^5^ of Mock- or Snail-B16F1 cells (clone Snail19-B16F1) in the suspension of 100 μl of DMEM. Mice were evaluated for tumor-sizes every couple of days (as indicated) and sacrificed 15 days later. Tumor volume was calculated using the formula: volume = *a* × *b*^2^ × 0.5, where *a* is the longest diameter and *b* the shortest [14]. Assays were performed in two independent experiments (n = 10 per arm).

### Statistical analysis

Results were expressed as mean ± S.D. Statistical significance between groups was calculated using unpaired Student's *t*-test. The *p* value < 0.05 was considered statistically significant. Significance was also estimated with block two-way ANOVA, using the cell line and the presence of lumican as grouping variables, and the post hoc Scheffe’s multiple comparisons test and contrast analysis.

## Results and Discussion

### Establishment and characterization of the Snail overexpressing B16F1 and HT29 cells

**Fig 1:**
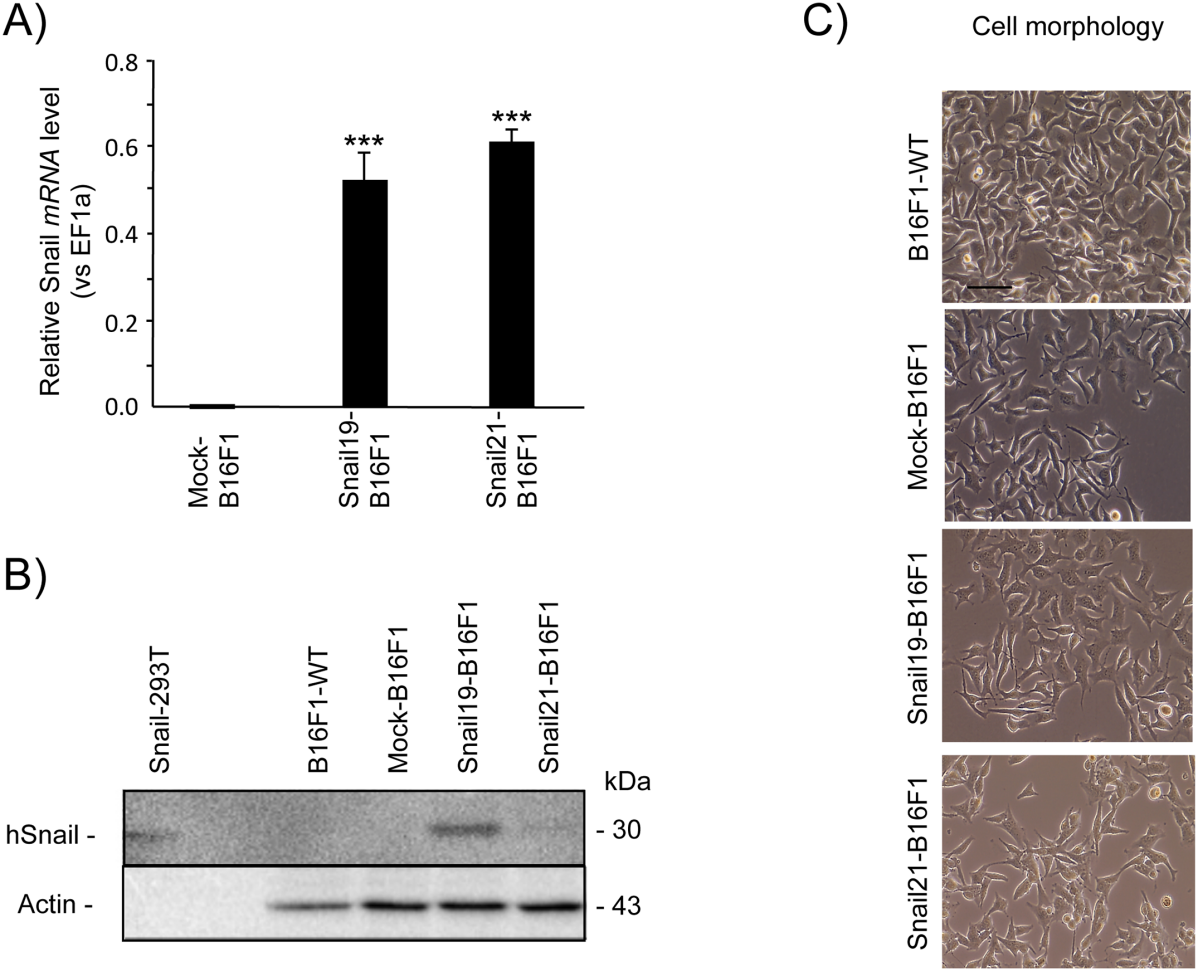
Characterization of Snail-B16F1 stable clones. Quantitative RT-PCR analysis of human snail (*SNAIL*) mRNA levels normalized to *EF1a*. Mean±SD, n = 4, ***p< 0.001. (B) Western blot analysis of Snail in B16F1-WT, in mock-B16F1 and in Snail overexpressing B16F1 cells (clones Snail19-B16F1—*high* expression of Snail and Snail21-B16F1—*low* expression of Snail). (C) Morphology (representative pictures) of B16F1-WT, mock-B16F1, and Snail-overexpressing B16F1 cells as observed by phase contrast microscopy (scale bar, 10 μm).

**Fig 2:**
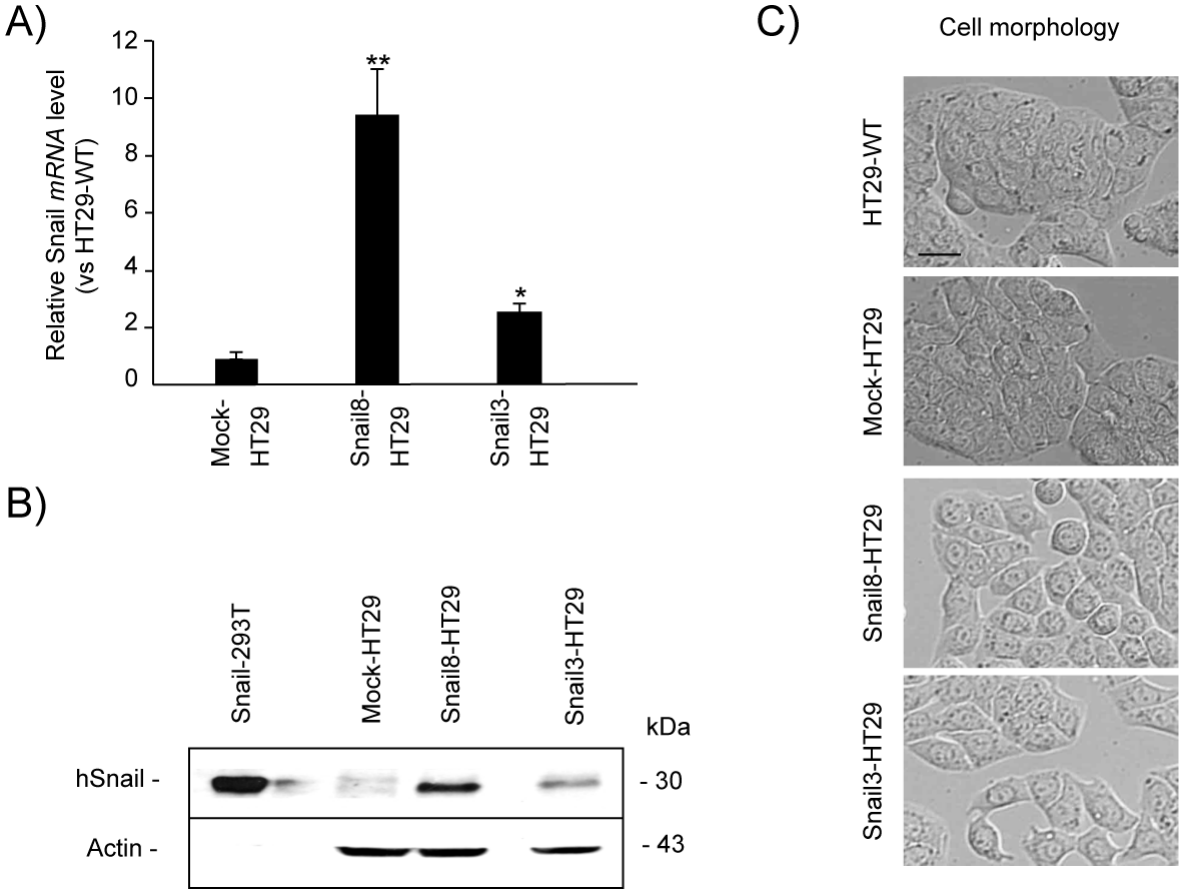
Characterization of Snail-HT29 stable clones. Quantitative RT-PCR analysis of human *SNAIL* mRNA levels normalized to the expression of *SNAIL* in HT29-WT. Mean±SEM, n = 8, *p< 0.02; **p< 0.01 (B) Western blot analysis of Snail in mock-HT29 and in Snail overexpressing HT29 cells (clones Snail8-HT29-*high* expression of Snail and Snail3-HT29 –*low* expression of Snail). (C) Morphology of HT29-WT, mock-HT29 and Snail-overexpressing HT29 cells as observed by phase contrast microscopy (scale bar, 10 μm).

Murine melanoma B16F1 cells and human colorectal adenocarcinoma HT29 cells were transfected with human *SNAIL* cDNA to establish stable clones exhibiting EMT-like features. After G418 selection, several stable Snail-positive clones of B16F1 and HT29 were obtained. Two clones of each line were selected for subsequent analysis: B16F1 clone 21 (Snail21-B16F1) and HT29 clone 3 (Snail3-HT29) with low Snail protein expression, and clones 19 (Snail19-B16F1) and 8 (Snail8-HT29), with demonstrated higher levels of Snail protein expression. As expected, human *SNAIL* expression was not detected in parental- or mock-transfected B16F1 cells, but was markedly increased in the selected clones both at the RNA and protein levels (Fig 1A and 1B, respectively). The stable Snail-B16F1 clone showed slight EMT-like changes with cellular characteristics such as spindle and dendritic shapes (Fig 1C). HT29-WT cells expressed small amount of Snail as detected by mRNA but it was not seen in immunoblotting on the protein level. However, relative mRNA levels in HT29 cells overexpressing Snail were significantly higher in both Snail3-HT29 and Snail8-HT29 clones (Fig 2A) with the amount of protein visibly increased (Fig 2B). Consistent with previous studies [45], Snail overexpression induced changes in cell phenotype in culture. The cells grew more scattered, acquired a spindle-shaped morphology and lost cell-to-cell contact (Fig 2C).

### Snail overexpression increases MMP-14 activity in B16F1 cells but not in HT29 cells

**Fig 3:**
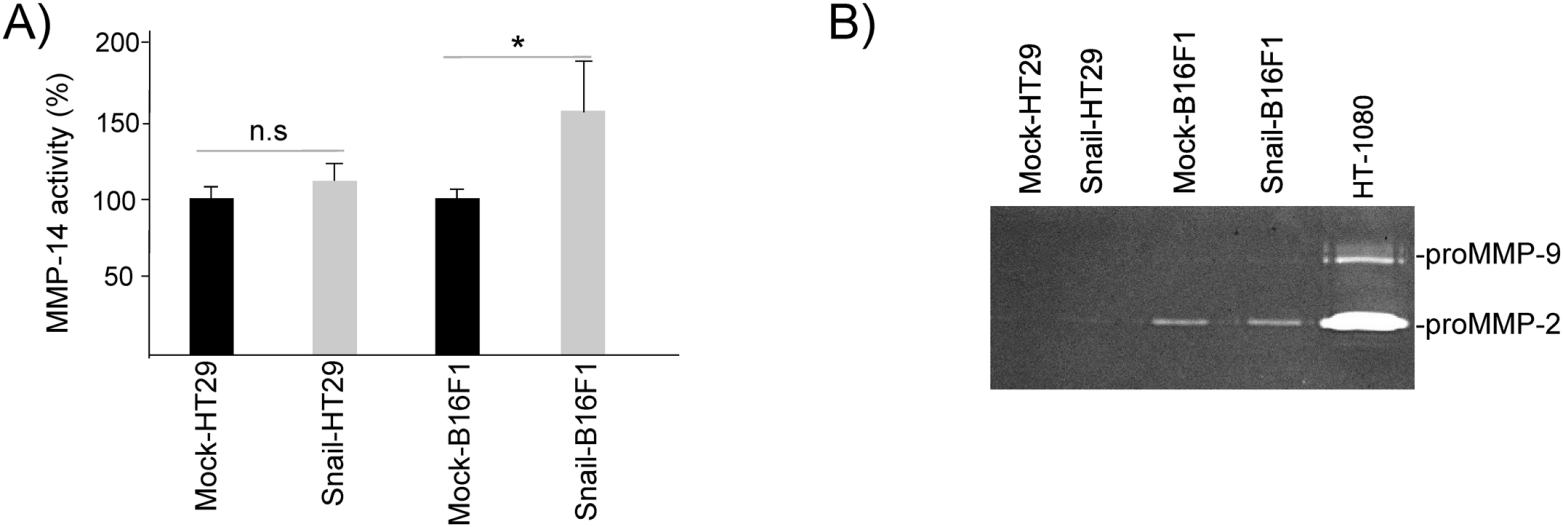
Effect of Snail overexpression and of lumican on MMP-2,-9 and MMP-14 expression and activity in B16F1 and HT29 cells. (A) MMP-14 activity in mock and Snail overexpressing B16F1 and HT29 cells (clones Snail19-B16F1 and Snail8-HT29). Mean±SEM, n = 3 experiments performed in duplicate. (B) MMP-2 and -9 expression in mock and Snail overexpressing B16F1 and HT29 cells (clones Snail19-B16F1 and Snail8-HT29). Cell-conditioned concentrated media were analyzed on SDS-polyacrylamide gels containing 1 mg/ml gelatin.

Since MMPs play an important role in cancer cell invasion, the activity of MMP-14 in control and Snail overexpressing -B16F1 as well as -HT29 cells was analyzed using the fluorimetric assay. Snail8-HT29 (high Snail expression clone) cells did not exhibit significant increase in MMP-14 activity in comparison with the mock-HT29 cells (Fig 3A). In contrast, MMP-14 activity was increased in Snail19-B16F1 cells (high Snail expression) by 1.6 fold in comparison with the mock-B16F1 cells (Fig 3A). In addition, Snail21-B16F1 cells, with low Snail protein expression, did not exhibit a significant increase in MMP-14 activity (S1 Fig). These results suggest a link between the level of Snail protein expression in B16F1 cells and MMP-14 activity. Moreover, the results indicate that Snail overexpression may affect MMP-14 activity differentially in each cell type. Several hypotheses can be raised to explain this. The effect of Snail on the activation of the MMP-14 by furin enzyme [46] might be different in B16F1 and HT29 cells. The distribution of the active form of MMP-14 on the cell membrane after Snail overexpression might also be different in the two cell types [47]. The mechanism of the differential regulation of MMP-14 activity between melanoma cells and colon cells by Snail requires further investigation. Interestingly, the overexpression of MMP-14 in colon adenocarcinoma cells was reported to be insufficient to increase experimental liver metastasis of human colon cancer cells [31] while it was sufficient for melanoma cells as our results suggest. Thus, MMP-14 might not play such a critical role in colon cancer cell development in contrast to melanoma progression where Snail regulates it. Cell migration through ECM is also regulated by the expression and activity of other matrix metalloproteinases. On the other hand, Snail overexpression did not influence either MMP-2 or MMP-9 activities in B16F1 and HT29 cells (Fig 3B). Since Snail affected MMP-14 activity in Snail-B16F1 cells but not in Snail-HT29, we pursued our investigations and the analysis of lumican effect in B16F1 cells but not in HT29 cells. The MMP-14 expression was also investigated using real-time PCR and Western immunoblotting in Snail- and mock-B16F1 cells. There was a non-significant increase in MMP-14 transcript in Snail overexpressing B16F1 cells. The level of MMP-14 transcript increased 1.12 fold in Snail19-B16F1 as compared to mock-B16F1 cells (S2 Fig).

### Lumican inhibits the Snail induced MMP-14 activity

**Fig 4:**
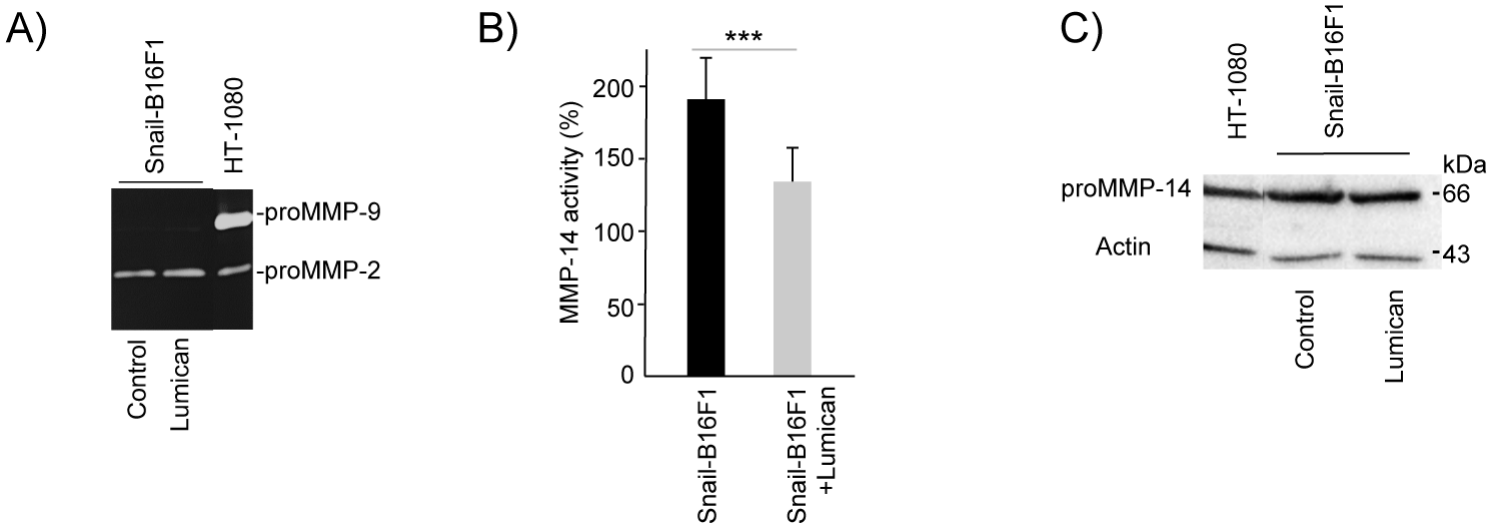
Effect of lumican on MMP-2,-9 and MMP-14 expression and activity in Snail-B16F1 cells. (A) Effect of lumican on MMP-2 and -9 activities in Snail overexpressing B16F1 cells (clone Snail19-B16F1). (B) Activity of MMP-14 in Snail-B16F1 cells (clone Snail19-B16F1) incubated 24h in the absence or in the presence of 100 nM lumican as described in Materials and Methods using fluorometric SensoLyte^™^ 520 MMP-14 Assay Kit. Data given as mean±SD, n = 6. Asterisk (*) indicates statistically significant differences between control and 100 nM lumican incubated Snail overexpressing B16F1 cells, (*** *p* < 0.001). Significance of differences estimated with block two-way ANOVA, using the cell line and the presence of lumican as grouping variables, and the post hoc Scheffe’s multiple comparisons test and contrast analysis. (C) Western blot analysis of MMP-14 expression in Snail overexpressing B16F1 cells (clone Snail19-B16F1) in the absence or in the presence of 100 nM lumican.

Lumican did not impair MMP-2 and MMP-9 activities in mock B16F1 cells (S3 Fig) and in Snail19-B16F1 cells (Fig 4A). In contrast, lumican was able to decrease significantly the elevated MMP-14 activity of the Snail19-B16F1 cells (Fig 4B). This is in agreement with our previous results that had shown inhibitory effects of lumican on B16F1 cells [30, 48]. Moreover, inactive pro-MMP-14 protein (~66kDa) was detected by Western immunoblotting in Snail19-B16F1 cells (Fig 4C), although the level of MMP-14 protein did not change in the presence of lumican (Fig 4C). Further studies are necessary for better understanding the mechanism of interaction between lumican and MMP-14 and the effect of Snail. Indeed, Snail has many targets. Besides E-cadherin, other direct targets for Snail repression have been identified including the epithelial Mucin-1 [49] and the components of the tight junctions claudins and occludin [50]. More importantly, Snail is upstream of signaling pathways involved in the degradation of the basement membrane and extracellular matrix by MMP-2 [47]. Snail is also a regulator of the expression of mesenchymal markers vimentin and fibronectin [49, 51] and other transcription factors such as ZEB-1 and LEF-1 [49]. Although a direct link between Snail expression and cytoskeletal proteins has not been reported, RhoB, a small GTPase involved in cytoskeletal actin rearrangements, lies downstream of Slug during chick neural crest delamination [52]. Since lumican was also shown to regulate the actin cytoskeletal network [17, 20, 21], lumican might interfere with the Snail/Slug effect in cytoskeletal actin rearrangements.

### Lumican inhibits the migration and cell growth of Snail overexpressing B16F1 cells *in vitro*

**Fig 5:**
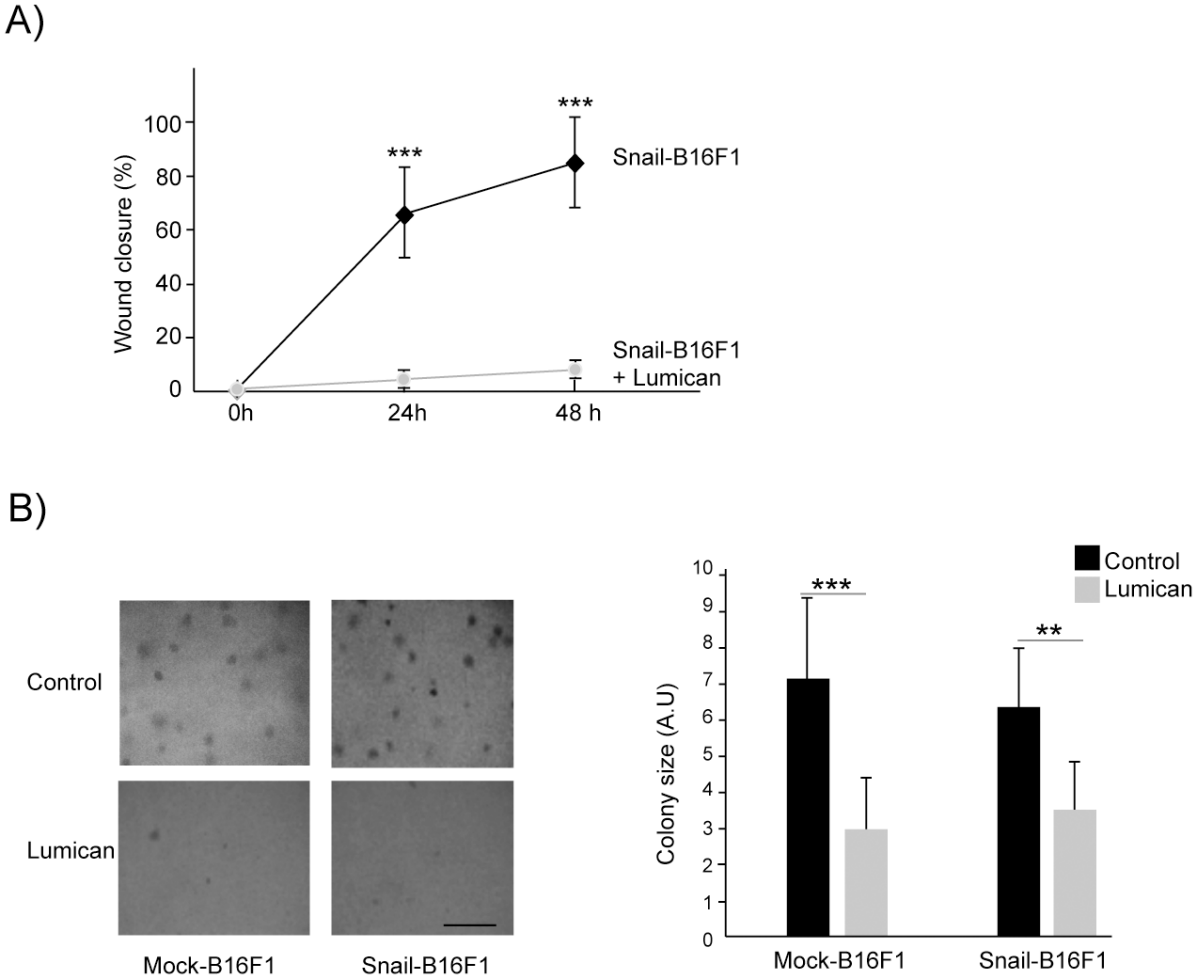
Lumican inhibits Snail overexpressing B16F1 cell functional properties. (A) Cell migration of Snail overexpressing B16F1 cells (clone Snail19-B16F1) in presence or absence of lumican (100 nM) after 24h and 48h; n = 2, ***p < 0.001 (B) Colony formation assay of mock and Snail overexpressing B16F1 cells (clone Snail19-B16F1) in absence or presence of lumican (100 nM); Representative images of cell colonies are displayed at the inserts 14 days after seeding. The quantification of the colony diameter was performed using Image Tool software. Graphs represent the mean size of 100 colonies ± SD from two independent experiments. (**p < 0.01, ***p< 0.001). Scale bar inserts: 200 μm.

Our previous studies demonstrated anti-migratory effect of lumican on the wild-type B16F1 cells [14, 18]. In the present report, we show that lumican significantly decreased the spontaneous cell migration of Snail19-B16F1 clone that expressed high amount of Snail protein. Cell migration was inhibited by almost 96% after 24 hrs (Fig 5A). In contrast, in Snail21-B16F1 clone that expressed low amount of Snail protein, lumican inhibited cell migration by around 30% (S4 Fig). Thus, the results suggest that B16F1 cell migration related to the high level of Snail expression is modulated by MMP-14 activity that may be inhibited by lumican. MMP-14 activity was increased in Snail19-B16F1 (Fig 3A), but not in Snail21-B16F1 (S1 Fig), and the Snail-induced MMP-14 activity was inhibited by lumican (Fig 4B).

The Snail induced EMT confers migratory and invasive properties to epithelial cells, critical for the generation of cells that originate at a distance from their final destination during embryonic development [53]. When the transition to a mesenchymal phenotype occurs in the adult, it is usually associated with pathological processes such as tumor progression [54]. Concomitant with the acquisition of motility, cells undergo dramatic changes in cell adhesion properties and cell shape [51–55]. In the present report, our results suggest that lumican inhibits B16F1 motility by Snail. The conversion to mesenchyme is correlated with a migratory cell phenotype and a profound reorganization of the cytoskeleton that may be incompatible with a highly proliferative state. To study the effects of lumican on cell proliferation in a semi-solid culture medium, the anchorage-independent growth of mock and Snail overexpressing B16F1 cells was investigated in soft agar cultures (Fig 5B). The sizes of the mock and Snail overexpressing B16F1 colonies were decreased by 57% and 46%, respectively (Fig 5B) after 14 days of cell culture in the presence of 100 nM lumican in comparison to control cultures grown in the absence of lumican. Altogether, these results show that lumican is able to inhibit the migration of the Snail-B16F1 cells. In addition, lumican is able to decrease B16F1 cell growth in soft agar.

### Lumican inhibits melanoma primary tumor growth of Snail overexpressing B16F1 cells *in vivo*

**Fig 6:**
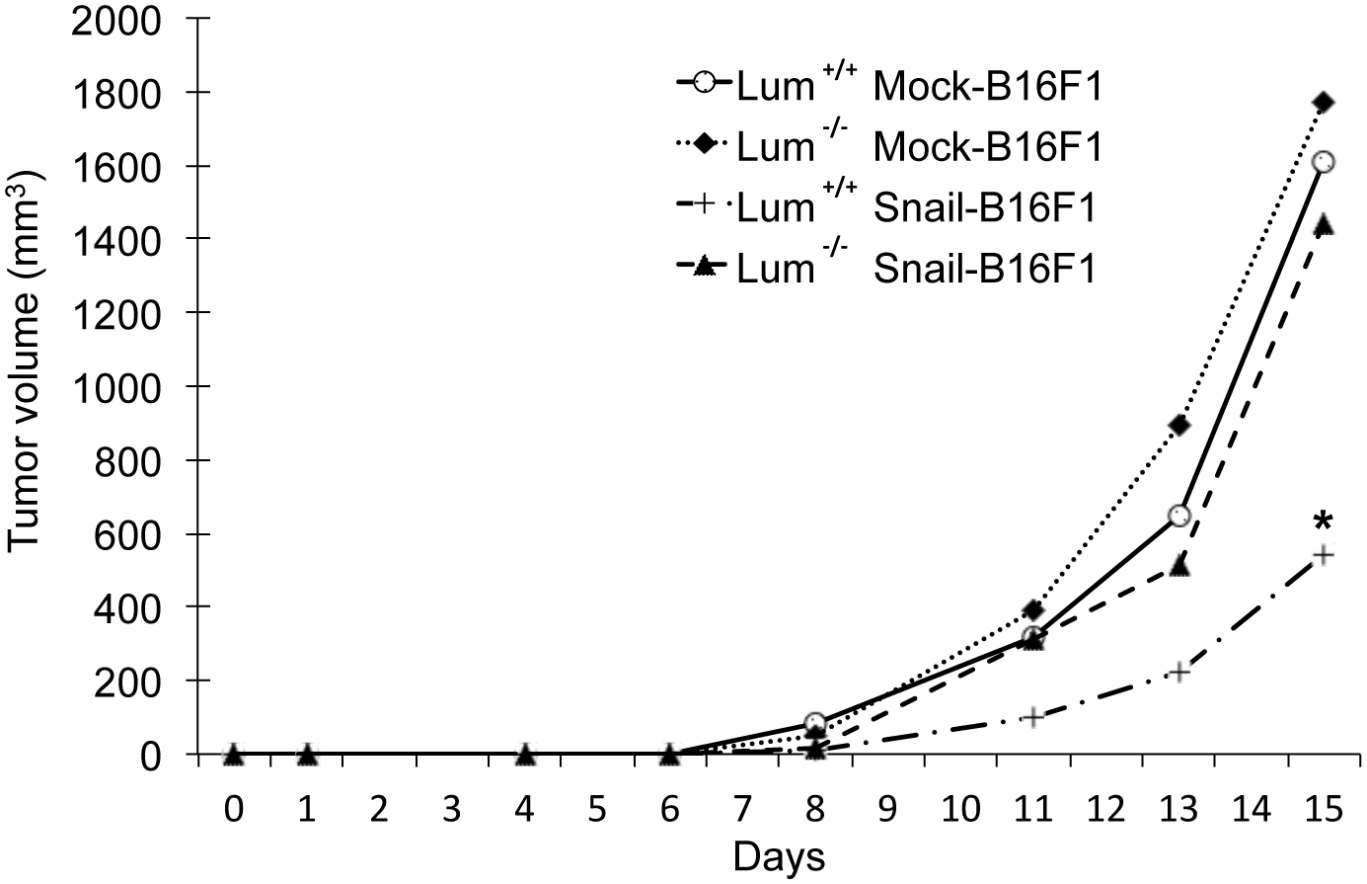
Lumican inhibits melanoma primary tumor growth of Snail overexpressing B16F1 cells *in vivo*. *Lum*^+/+^ and *Lum*^-/-^ mice on the C57Bl/6 background were injected subcutaneously with 2.5 x 10^5^ of B16F1 cells, mock or Snail overexpressing B16F1 cells (clone Snail19-B16F1). The kinetic of melanoma primary tumor growth is displayed in volume (mm^3^) till day 15 when mice were sacrificed. * *p* < 0.05: significantly different from mock-transfected B16F1 cell tumors, Open circle: wild type C57Bl/6 mice injected with mock-B16F1 cells (n = 10); Diamond: lumican deleted C57Bl/6 mice injected with mock B16F1 cells (n = 10); Open square: wild type C57Bl/6 mice injected with Snail overexpressing B16F1 cells (n = 10); Triangle: Lumican deleted C57Bl/6 mice injected with Snail overexpressing B16F1 cells (n = 10).

To study the potential inhibitory effect of lumican *in vivo*, Snail-B16F1 (clone Snail19-B16F1 line) and mock B16F1 cells were implanted subcutaneously into wild type (*Lum*^+/+^) and lumican-deleted (*Lum*^-/-^) C57BL/6 mice [4]. Melanoma primary tumors were detected 8 days after B16F1 cell injection (Fig 6). Snail-B16F1 tumors grew more slowly than mock-B16F1 tumors, which is probably due to the inhibitory effect of Snail on cell proliferation [56]. Indeed, Snail has been shown to regulate cell-cycle progression and survival by regulating components of the early to late G1 transition and the G1/S checkpoint, including the repression of *CyclinD2* transcription and the increase in p21/Cip1. More importantly, in the current study, 15 days after injection of cells, the volume of tumor induced by Snail overexpressing B16F1 cells was drastically decreased in wild type *Lum*^+/+^ mice as compared to *Lum*^-/-^ mice (Fig 6). This result suggests that the endogenous lumican of wild type mice is an important inhibitor of Snail-B16F1 cell tumor development. The inhibitory effect of endogenous lumican on mock-B16F1 cell tumor development is less visible. These results underscore the importance of Snail in the anti-tumor effects of lumican. These *in vivo* studies are in agreement with our *in vitro* results showing a decrease of colony formation, cell migration, and MMP-14 activity in Snail overexpressing cells in presence of lumican. Additional studies are required to better understand the molecular mechanisms of action between Snail and lumican.

## Supporting Information

S1 Fig MMP-14 activity in mock and Snail overexpressing B16F1 clones. The measurements were performed as described in Materials and Methods. Mean±SEM, n = 3 experiments performed in duplicate.
(TIFF)

S2 Fig MMP-14 expression in mock and Snail-B16F1 cells (clone 19). Mean±SEM, n = 3 experiments performed in duplicate. n.s = non-significant.
(TIFF)

S3 Fig Effect of lumican on MMP-2 and MMP-9 activities in mock B16F1 cells. Cell-conditioned concentrated media were analyzed on SDS-polyacrylamide gels containing 1 mg/ml gelatin.
(TIFF)

S4 Fig Cell migration of Snail overexpressing B16F1 cells (clone 21). Cell migration of Snail21-B16F1 in presence or absence of lumican (100 nM) after 24h and 48h; n = 2, *p < 0.05.
(TIFF)

S1 Table List of primers used for quantitative real time PCR reaction. *SNAI1*: snail family zinc finger 1; *MMP-14*: matrix metalloproteinase-14; *EF1a*: elongation factor 1 alpha 1; *GAPDH*: glyceraldehyde-3-phosphate dehydrogenase.
(DOCX)

## Abbreviations

SLRP: small leucine-rich proteoglycan
ECM: extracellular matrix
EMT: epithelial-mesenchymal transition
LRR: leucine rich repeats
MMP: matrix metalloproteinase
FRET: fluorescence resonance energy transfer
GAG: glycosaminoglycan
