# Supplementary figures and images for: Lumican Inhibits SNAIL-Induced Melanoma Cell Migration Specifically by Blocking MMP-14 Activity
Source: PLoS One. 2016 Mar 1;11(3):e0150226. doi: 10.1371/journal.pone.0150226 (PMC4773148; doi:10.1371/journal.pone.0150226)

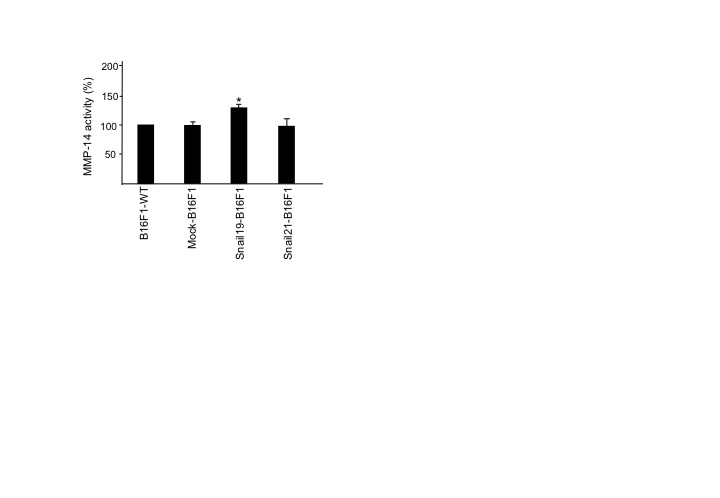

Supplement: S1 Fig — The measurements were performed as described in Materials and Methods. Mean±SEM, n = 3 experiments performed in duplicate. (TIFF) [file pone.0150226.s001.tiff]

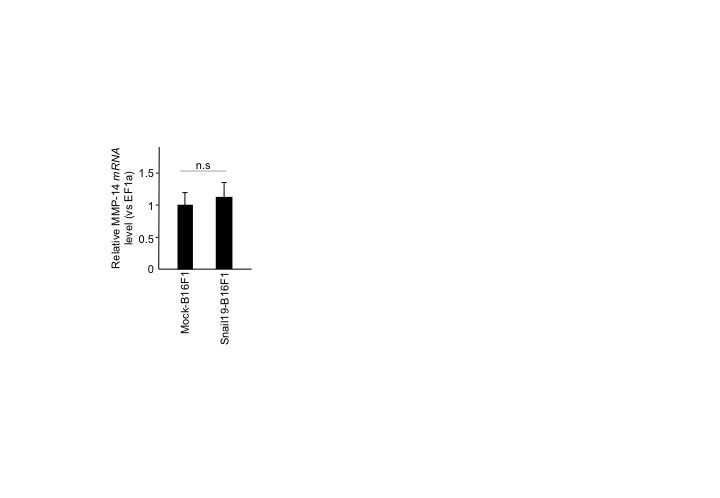

Supplement: S2 Fig — Mean±SEM, n = 3 experiments performed in duplicate. n.s = non-significant. (TIFF) [file pone.0150226.s002.tiff]

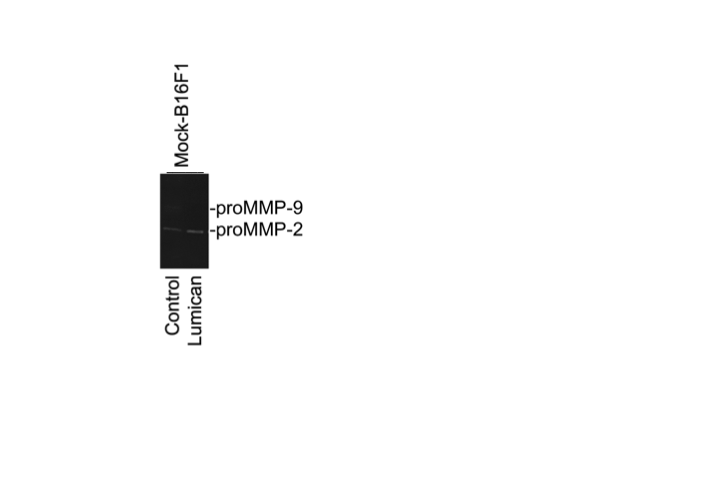

Supplement: S3 Fig — Cell-conditioned concentrated media were analyzed on SDS-polyacrylamide gels containing 1 mg/ml gelatin. (TIFF) [file pone.0150226.s003.tiff]

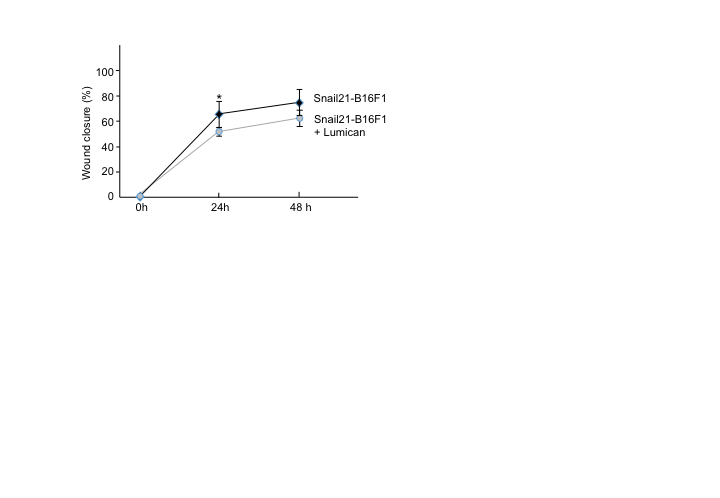

Supplement: S4 Fig — Cell migration of Snail21-B16F1 in presence or absence of lumican (100 nM) after 24h and 48h; n = 2, *p < 0.05. (TIFF) [file pone.0150226.s004.tiff]
