# Supplementary material for: Lumican Inhibits SNAIL-Induced Melanoma Cell Migration Specifically by Blocking MMP-14 Activity
Source: PLoS One. 2016 Mar 1;11(3):e0150226. doi: 10.1371/journal.pone.0150226 (PMC4773148; doi:10.1371/journal.pone.0150226)
Supplement: S1 Table — SNAI1: snail family zinc finger 1; MMP-14: matrix metalloproteinase-14; EF1a: elongation factor 1 alpha 1; GAPDH: glyceraldehyde-3-phosphate dehydrogenase. (DOCX) [file pone.0150226.s005.docx]

S1 Table. List of primers used for quantitative real time PCR reaction.

| **Primer** | **Sequence (5'→3')** | **Amplicon size (bp)** | **Accession number** |
| --- | --- | --- | --- |
| SNAI1 | F-GTCGCAGGACTCTAATCCAGA  r-ATCTCCGGAGGTGGGATG | 84 | NM_005985 .3 |
| MMP-14 | F- CGGGTGAGGAATAACCAAGT  R- CCAGAAGAGAGCAGCATCAA | 237 | NM_004995.2 |
| EF1a | F- CTGGAGCCAAGTGCTAATATGCC  R- CCAGGCTTGAGAACACCAGTC | 222 | NM_001402 |
| GAPDH | F- ACGGATTTGGTCGTATTGGG  R- TGATTTTGGAGGGATCTCGC | 230 | NM_002046.4 |

SNAI1: snail family zinc finger 1; MMP-14: matrix metalloproteinase-14; EF1a: elongation factor 1 alpha 1; GAPDH: glyceraldehyde-3-phosphate dehydrogenase.
